# The Physiotherapy Intervention for Shoulder Pain in Patients Treated for Breast Cancer: Systematic Review

**DOI:** 10.7759/cureus.6416

**Published:** 2019-12-18

**Authors:** Andrea Giacalone, Paola Alessandria, Enzo Ruberti

**Affiliations:** 1 Department of Industrial Engineering, University of Tor Vergata, Roma, ITA; 2 Department of Physical Therapy, University of Turin, Turin, ITA; 3 Department of Neurology, Sapienza University, Roma, ITA

**Keywords:** upper extremity pain, physical therapy, breast cancer, shoulder

## Abstract

Pain and joint dysfunction of the upper limb following treatment for mammary carcinoma are defined as the most frequent side effects of surgery for mammary carcinoma by calculating a prevalence range in the USA that varies between 12% and 51% with regard to pain and between 1.5% and 50% for joint dysfunction. This objective of this study was to describe physiotherapy interventions that demonstrate efficacy based on the type of pain present in patients with shoulder pain who have been treated for breast cancer.

We conducted a search for relevant publications in the last 10 years (2009-2019) on the PubMed, Medline, CINAHL, and Cochrane databases. The articles obtained were selected on the basis of correlation criteria, with themes concerning shoulder pain after mammary carcinoma and physiotherapy performed to treat this pain.

Studies suggest treating musculoskeletal pain through active exercises, joint and tissue mobilization techniques, with accessory joint mobilization and neurodynamics performed by experienced physiotherapists. In order to treat radio-induced and drug-induced neuropathic pain, both aerobic and strengthening exercises are supervised by an experienced physiotherapist and carried out twice weekly for a total of 150 minutes of exercise. Finally, the treatment suggested to deal with chronic pain uses a multidisciplinary approach, including pain education interventions, pharmacological interventions, psychological interventions, and physiotherapy interventions.

To conclude. the classification of different types of shoulder pain following mammary carcinoma treatments requires specific and targeted physiotherapy interventions in which active therapeutic exercise has a central role.

## Introduction and background

Breast cancer is the most common malignancy in women and the main cause of morbidity and mortality. In fact, there are 1.67 million new cases of breast cancer in the world each year and 458,000 annual deaths related to it [1]. About 89% of women with breast cancer survive at least five years after treatment, but side effects may persist for months or years following surgery [2]. Pain and joint dysfunction of the upper limb are defined as the most frequent side effects by calculating a range of prevalence that varies between 12% and 51% for pain and between 1.5% and 50% for joint dysfunction [3-4].

In the study, there is a greater risk of shoulder pain presenting following treatments for mammary carcinoma in patients with a lower value on the Karnofsky Performance Status scale and a higher score on the Social Communication Questionnaire [5]. Furthermore, the patient's poor general physical condition, low socioeconomic status, non-Caucasian race, and, finally, a low score on the Quality of Life scale increase this probability. The meta-analysis study indicates that the predictors of pain are the presence of preoperative shoulder pain and the younger age of patients [6]. A high percentage of patients experiencing moderate pain have undergone mastectomy and extensive axillary dissection and present a greater number of removed lymph nodes, positive lymph nodes, and drains inserted during surgery. There is a likelihood of greater complications in patients complaining of moderate pain in the sub-acute period following surgery.

In many situations, together with the preoperative assessment, patients are given an educational booklet with a summary of the advice and precautions to be implemented in the period following the surgical operation, so as to have a reinforcement role on the information obtained during the preoperative meeting [7-8]. McNeely's study, in particular, suggests that a supervised rehabilitation intervention is preferable to the standard one, which instead uses an informative booklet, a written educational package, or unstructured or non-individualized exercises [8]. In fact, personalized physiotherapy demonstrates the ability to reduce complex problems related to the postsurgical morbidity of mammary carcinoma thanks to the early identification of symptoms. It is, therefore, essential to recognize the most effective treatment based on the pain presented by the patient. Shoulder pain following treatment for mammary carcinoma can, in fact, be divided into musculoskeletal nociceptive type pain, drug-induced neuropathic and radio induced types and, finally, as chronic persistent pain [9-10].

Musculoskeletal nociceptive pain arises from the fact that treatments for breast cancer directly involve the neuromusculoskeletal tissues of one or more limb areas, causing shoulder pain, joint limitation, and hyposthenia [11-12]. The population of breast cancer operated patients may present a reduced variety of motor strategy, producing movements with increasingly reduced ranges reporting a subacromial impingement and, therefore, pain [3,11,13-18]. In Ebaugh's study, it is stated that postural pain, scar formation, and protection posture cause a shortening of the small pectoral and large pectoral muscles [12]. It can cause a posture characterized by the depression and antepositioning of the shoulder. Radiation therapy and the subsequent tissue fibrosis can also exacerbate this misalignment, which would result in an incorrect relationship both between the scapula and the chest wall and between the humeral head and the glenoid fossa. These structural changes can become chronic in the musculoskeletal tissues related to the shoulder, eventually causing pain.

Neuropathic pain syndromes can be divided into neuropathic pain related to cancer, neuropathic pain caused by drug-induced treatment, and neuropathic pain associated with cancer [9]. Postmastectomy pain syndrome is a common example of neuropathic pain induced by cancer therapy. Radiotherapy can also cause neuropathic pain: in fact, it can affect the surrounding structures, including the nerves and the neural plexus [10]. Finally, neuropathic pain can be drug-induced by taking aromatase inhibitor drugs. They are the most frequently used hormonal treatment for the treatment of hormone-responsive tumors, which boasts the greatest scientific evidence [19].

However, about 50% of patients taking anti-aromatases report widespread musculoskeletal pain, defined as "aromatase inhibitor musculoskeletal syndrome," which persists even after stopping the drug. It is manifested by arthralgia, osteoporosis, and the risk of fractures. The onset of arthromial pain is reported as the most frequent cause of treatment suspension [20-21]. Lipps's study analyzes the data for radiotherapy side effects [22]. Although radiotherapy is an effective treatment for mammary carcinoma, some patients may report comorbidity in the ipsilateral shoulder. Fatigue, skin reactions, and pain are the most frequently reported side effects; in particular, over 60% of patients experience pain at the end of treatment and, in 4% of cases, pain is reported to be severe or very severe. Radiation therapy of muscles and connective tissues within the shoulder joint can lead to the formation of fibrosis and atrophy and, therefore, to pain.

Finally, chronic pain occurs in a proportion of up to 50% of patients six months after surgery [23]. Breast cancer survivors with persistent breast pain have significantly high levels of depressive symptoms, increased apprehension of pain, and increased anxiety as compared to patients who do not have persistent breast pain or women who have no history of cancer [24]. Inadequately managed postoperative pain is a relevant risk factor in chronic pain syndrome. Despite an 83% survival rate, it is estimated that between 25% and 60% of breast cancer survivors experience post-surgical persistent pain, associated with reduced quality of life and motor dysfunction [9]. Some patients with chronic pain show features that suggest central pain sensitization, a process characterized by a generalized hypersensitivity of the somatosensory system, defined as an amplification of the neural signal at the level of the central nervous system [25].

Objectives

The purpose of this research is to select articles related to physiotherapy interventions in relation to the topic of shoulder pain in women treated for breast cancer and to identify the most effective physiotherapy interventions according to evidence-based medicine (EBM) based on the classification of the different types of pain typically described as a result of treatments for mammary carcinoma.

## Review

Methods

Research Question

What are the physiotherapy interventions with the most efficacy for the treatment of shoulder pain in women treated for mammary carcinoma?

Eligibility Criteria

*P (population): Patients treated for mammary carcinoma with shoulder pain. Treatments include surgical procedures, such as mastectomy, axillary lymph node dissection, sentinel lymph node biopsy, and taking anti-aromatase drugs and radiotherapy. *I (intervention): Physiotherapeutic interventions recognized by scientific evidence useful for the reduction of pain presented by patients following treatments for mammary carcinoma. *C (comparison): Patients who receive only standard assistance/usual care. In many studies, it is represented by preoperative counseling followed by the delivery of informative booklets. *O (results): Definition of rehabilitative intervention demonstrating efficacy based on the classification of shoulder pain presented by patients following treatments for mammary carcinoma.

The linguistic restriction was chosen because all the studies were in English and the temporal restriction of the studies was of the last 10 years (2009-2019) in which it was possible to read the abstract and then the full text.

The articles obtained were selected on the basis of correlation criteria with themes concerning shoulder pain following mammary carcinoma and physiotherapy performed to treat this pain.

Sources of Information and Research

The search for relevant publications of the last 15 years (2004-2019) was carried out through the use of the PubMed, Medline, CINAHL, and Cochrane databases. In particular, on PubMed, Medline, and CINAHL, the research was conducted using the following Boolean operators:

(((("Upper Extremity"[Mesh] OR upper Extremit* AND pain OR Upper Limb* AND pain))) OR ("Shoulder Pain"[Mesh] OR shoulder pain*)) AND ((("Breast Neoplasms"[Mesh] OR ((Breast OR (Cancer OR Neoplasm* OR malignanc* OR tumor* OR tumour*)))) AND ("Shoulder"[Mesh] OR shoulder*))) AND ("Physical Therapy Modalities"[Mesh] OR Physical Therapy Modalit* OR Physical Therapy Techniqu* OR Physiotherap*)

In the Cochrane Library, a mesh search was performed with the following free words:

breast cancer or breast neoplasm or breast carcinoma or breast tumor and physiotherapy or physical therapy or physiotherapist or physical therapist and shoulder pain

Study Selection

The total number of articles identified through mesh research is 682 studies. Two-hundred ninety-nine articles were excluded because they did not meet the inclusion criteria, 258 articles were excluded after reading the abstract, and a further 112 after reading the full article text. Thirteen articles were found to be useful and relevant to the research question, in particular, four systematic reviews, three narrative reviews, three randomized controlled trials, two randomized controlled pilot studies and one uncontrolled trial (Table 1).

**Table 1:** Literature search of evidence-based physical therapy interventions in breast cancer patients with shoulder pain

IDENTIFICATION	SCREENING	ELIGIBILITY	INCLUDED
Articles identified through the research: Pubmed, Medline, Cinahl and Cochrane (N=682)	Inclusion criteria: studies of the last 10 years, English language, possible reading of the abstract (N=283)	Abstract assessed for eligibility (N=125)	Full-text studies included in qualitative summary (N=13)
	Excluded studies (N=299)	Excluded studies (N=258)	Excluded studies (N=112)

Shoulder pain was then classified as musculoskeletal nociceptive pain, drug-induced and radio-induced neuropathic pain, and chronic pain. Based on the type of pain, the recommended physiotherapy treatment is identified according to EBM.

Risk of Bias Across Studies

The possibility of bias for structural variability of the studies considered and for the subjective evaluation of the inclusion of the studies following their full-text reading. Furthermore, the nature of the treatments made it impossible to carry out a blind study both for patients and for the health care professionals who apply them. Finally, the three narrative reviews cannot be evaluated according to this scheme.

Results

The data of the recent literature suggest that multifactorial physiotherapy (e.g. stretching and exercises) and active exercises are effective for the treatment of postoperative pain and reduced joint following surgery for mammary carcinoma [4]. High-quality studies state that carefully monitored exercise prescriptions and analysis of persistent upper limb dysfunctions are necessary [8,26]. In fact, perspective surveillance and target physiotherapy, as compared to education, show that target physiotherapy is more suitable for women with mammary carcinoma due to the early identification of upper limb morbidity. In Rafn‘s study, in fact, the patients treated for mammary carcinoma are divided into two groups with randomized modality: one group is followed with prospective surveillance and target physiotherapy and one group is followed with standard care, consisting of preoperative education and the delivery of a booklet of exercises to be performed at home [7]. Perspective surveillance consists of the quarterly monitoring of the patients, assessing the shoulder articulation variables, strength, volume, and functionality of the upper limb. If problems are identified to the upper limb, the patients are sent to perform physiotherapy, which, in this way, becomes the "target." At 12 months, patients treated with education alone presented more complex morbidity than the group treated with target physiotherapy. In the postoperative period, it is important to consider the early implementation of the exercise, although this approach should be cautious to avoid complications at drainage points [8]. For the treatment of musculoskeletal nociceptive pain, the use of manual therapy and complex decongestive physiotherapy in elderly patients operated on for mammary carcinoma was evaluated as effective. The use of manual therapy can, indeed, improve the feeling of pain and increase the range of motion (ROM) of the shoulder [27]. In the same way, the use of myofascial inductions can be moderately effective [28]. However, the most solid positive effects are identified in therapeutic exercise for the reduction of the pain and in yoga with effects on the state of anxiety [29]. Active exercises show that the best results of joint recovery are achieved between the 28th and 42nd postoperative days [4]. In the sub-acute phase, the muscle groups that need major reinforcement are those of the rotator cuff but also the serratus anterior, upper and middle trapezius, the rhomboids, the biceps femoris, and the small and large pectoralis [14]. Training can start with exercises that use elastic bands, always taking great care of maintaining the correct posture. It is recommended to carry out 10 to 15 repetitions for each exercise for two sets, twice a week. The best way to start is to begin with more repetitions and less resistance and gradually increase resistance by reducing repetitions. Later, we will move to using machinery to increase reinforcement [30]. In addition to selective reinforcement exercises, control exercises and neuromuscular proprioceptive facilitation are recommended for the restoration of the correct scapulohumeral rhythm [8,31]. The most effective treatment with regard to neuropathic pain given by taking aromatase inhibitors is found in the 12-month extended exercise, which led to an improvement in arthralgia in previously inactive mammary carcinoma survivors compared with patients who received standard informational treatment [21]. The treatment proposed in Irwin’s study foresees the execution of exercises of both aerobic and reinforcement types, performed with supervision and carried out twice weekly for a total of 150 minutes [21]. It should also be noted that, although some benefits were found after three months of exercise, the most obvious improvements occurred after 12 months, reporting a follow-up with a reduction in pain of 29%. For the treatment of arthralgia associated with anti-aromatase drugs, the efficacy of the neurotaping used for five weeks was also observed. The result obtained was the subjective reduction of pain [19]. Regarding the neuropathic pain given by radiotherapy, the treatment was evaluated by means of a moderate intensity, supervised therapeutic exercise. In addition to improving the sensation of pain, statistically significant improvements were calculated in the World Health Organization Quality of Life (WHOQOL), brief fatigue inventory (BFI), and shoulder ROM [32]. The studies of Nijs and Geneen provide a general overview regarding a greater understanding of the psychosocial mechanisms of chronic pain in patients treated for breast cancer and deepen on how to set up exercise with patients having different side effects [10,33]. Lack of sleep induces a low-grade inflammatory response that can lead to pain sensitization typical of chronic pain. Pain education is an effective strategy for treating chronic cancer-related pain with few side effects. Similarly, cognitive-behavioral therapy for sleep problems, stress management, and active exercise can improve sleep in these patients. The rehabilitative approach which includes sleep management, functional targets, and graded therapeutic exercise with selective muscle strengthening, are often associated together with psychological strategies for the management of pain, anxiety, and depression. Physiotherapeutic activity, therefore, aims to assist the psychologist in the active management of pain through the positive experience of guided exercise and muscle activation, also associated with the production of oxytocin. The physical exercise associated with pain education and cognitive-behavioral therapy is, therefore, effective for the reduction of persistent pain. Indeed, there are favorable effects of therapeutic exercise on the reduction of chronic pain and an increased physical function, although the effects are mild to moderate. Finally, variable effects for psychological functions and quality of life have been calculated. See Tables 2-3.

**Table 2:** Risk-of-bias assessment using the Cochrane tool Criteria Items: 1, Was the method of randomization adequate? 2, Was the treatment allocation concealed? 3, Was the patient blinded to the intervention? 4, Was the care provider blinded to the intervention? 5, Was the outcome assessor blinded to the intervention? 6, Was the dropout rate described and acceptable? 7, Were all randomized participants analyzed in the group to which they were allocated? 8, Are reports of the study free of suggestion of selective outcome reporting? 9, Were the groups similar at baseline regarding the most important prognostic indicators? 10, Were co-interventions avoided or similar? 11, Was the compliance acceptable in all groups? 12, Was the timing of the outcome assessment similar in all groups? N, no; U, unsure; Y, yes

Article	1	2	3	4	5	6	7	8	9	10	11	12	Score
De Groef A, et al.	Y	Y	N	N	Y	Y	U	Y	Y	Y	Y	N	8/12
Rafn BS, Hung, et al.	Y	Y	N	N	Y	Y	Y	Y	Y	Y	Y	Y	10/12
McNeely, Campbell, et al.	N	N	N	N	Y	Y	Y	Y	Y	U	Y	Y	7/12
Conejo I, Pajares, et al.	Y	Y	N	N	N	Y	Y	Y	Y	Y	Y	Y	9/12
Irwin ML, Cartmel B, et al.	Y	Y	N	N	N	Y	Y	Y	Y	U	Y	Y	8/12
Tunay VB, Akbayrak, et al.	N	Y	N	N	Y	Y	Y	Y	Y	Y	Y	Y	9/12
Castro-Martin E, et al.	Y	U	N	U	Y	Y	Y	Y	Y	Y	Y	Y	9/12
Olsson Möller U, Beck I, et al.	N	U	U	U	U	Y	Y	Y	Y	U	Y	Y	6/12
Hwang JH, Chang HJ, et al.	Y	N	N	N	U	Y	Y	Y	U	U	Y	Y	6/12
Geneen LJ, Moore RA, et al.	Y	Y	N	N	Y	Y	Y	Y	N	U	Y	N	7/12

**Table 3:** Summary of studies that investigated the evidence-based physical therapy interventions in breast cancer patients with shoulder pain RCT: randomized controlled trials; ROM: range of motion

AUTHOR	YEAR	OBJECTIVE	SAMPLE	MEANS	RESULTS
De Groef A, Van Kampen M, Dieltjens E et al.	2015	Systematic review to evaluate the effectiveness of various physiotherapy modalities and their timing for the recovery of articularity in the upper limb and pain management following treatment for mammary carcinoma. Modalities include passive mobilization, passive stretching, myofascial techniques and active exercise	18 RCT		Multi-factor physiotherapy (e.g. stretching and exercises) and active exercises have proven to be effective for the treatment of postoperative pain and reduced articulation following surgery for mammary carcinoma
Geneen LJ, Moore RA et al.	2017	Cochrane Systematic Review to determine the effectiveness of different activities and therapeutic exercises for the reduction of chronic pain and its impact on function, quality of life and to verify the evidence for any side effect associated with physical activity and therapeutic intervention.	21 reviews with 381 studies. Of these, 264 studies were used for qualitative analysis.	AMSTAR	There are some favorable effects of therapeutic exercise on pain relief and increased physical function, although these effects are mild to moderate. There are variable effects for psychological functions and quality of life. Physical activity and therapeutic exercise are interventions with few side effects.
McNeely M. L., Campbell et al.	2010	Cochrane systematic reviews of RCTs to evaluate the efficacy and safety of therapeutic exercise for the prevention or improvement of upper limb dysfunctions due to treatment for mammary carcinoma.	10 of 24 RCTs		Exercises can bring significant clinical improvement in shoulder joint in women with mammary carcinoma. In the postoperative period, it is important to consider the early implementation of the exercise, although this approach should be cautious to avoid complications at drainage points. High-quality studies state that carefully monitored exercise prescriptions and analysis of persistent upper limb dysfunctions are necessary.
Amatya B., Khan et al.	2017	Review to optimize post-acute care in rehabilitative mammary carcinoma survivors	Narrative review		The rehabilitation intervention should be considered early in order to maintain functional capacity and to reduce the risk of losing important skills or independence and should be individualized according to the stage of illness, functional deficits, personal requests, and specific objectives. Multidisciplinary rehabilitation and uni-disciplinary interventions, such as physiotherapy, have been shown to be useful in reducing disability and improving participation and quality of life.
Tunay V. B., Akbayrak et al.	2012	Evaluate the effects of manual therapy and therapeutic exercise on shoulder function, pain, and lymphedema in elderly patients with mammary carcinoma	40 women with radical mastectomy	goniometer, VAS, metro, DASH, SF-36,	The study demonstrates the effectiveness of manual therapy and complex decongestive physiotherapy in elderly patients operated for mammary carcinoma. The use of manual therapy can reduce pain and improve shoulder ROM.
Irwin, M. L., Cartmel, B et al.	2015	Randomized exercise trial to calculate the impact of therapeutic exercise versus standard treatment in women with anti-aromatase-induced arthralgia	121 women surviving mammary carcinoma with AI arthralgia, of which 61 treated with exercises of increasing intensity	Brief Pain Inventory (BPI), Western Ontario and McMaster Universities Osteoarthritis (WOMAC) index, Disabilities of the Arm, Shoulder, and Hand (DASH) questionnaire, dynamometer.	Exercise maintained for 12 months led to an improvement in arthralgia in previously inactive mammary carcinoma survivors.
Conejo I., Pajares et al.	2018	Pilot RCT study to evaluate the efficacy of neurotaping in the treatment of arthralgia associated with anti-aromatases in women treated for mammary carcinoma	40 women with endocrine therapy after mammary carcinoma	profile of mood state (POMS), fatigue evaluation (Quickpiper), quality of life (EORTC QLQ-C30), dynamometer, VAS, Spine Functional Index (SFI), Upper Limb Functional Index (ULFI), Backache Disability Index (BADIX)	After 5 weeks of neuromuscular taping, patients treated with neuromuscular taping have improved their pre-existing musculoskeletal symptoms, particularly in the subjective sensation of pain.
Casla S., Hojman et al.	2015	Provide a general overview of the benefits of therapeutic exercise on breast cancer patients and on recommendations for setting exercise with patients having different side effects	Narrative review		Exercise is a global intervention that improves the physical, psychological and psychosocial aspects of patients with mammary carcinoma. Appropriate exercises can, in fact, reduce the side effects and other comorbidities that patients can present in the long term, thus improving the quality of life and their survival.
Nijs J., Leysen et al.	2018	Trying to explain how increased knowledge in neuroscience can improve adult rehabilitation following cancer treatment within an evidence-based perspective	Narrative review		Pain education is an effective strategy for treating cancer-related pain. Furthermore, the neuro-immunological knowledge of how stress affects pain emphasizes the importance of integrating stress management within the rehabilitation approach. Lack of sleep induces a low-grade inflammatory response that can lead to pain sensitization typical of chronic pain. Cognitive behavioral therapy for sleep problems, stress management, and active exercise can improve sleep in these patients. Exercise is effective for pain relief following cancer and can reduce the pain associated with hormonal cancer treatments.
Rafn B. S., Hung et al.	2018	Randomized controlled single-blind pilot studies to assess prospective surveillance and target physiotherapy compared to education on the prevalence of arm morbidity following mammary carcinoma surgery	41 women operated for mammary carcinoma, of which 21 followed target physiotherapy and 20 with education	Hand-held dynamometer (HHD; MicroFET2 Hand Held Dynamometer; Hoggan Scientific, Salt Lake City, USA) and HHD hand-held dynamometer (Jamar Plus + Digital Hand Dynamometer; Lafayette Instrument, Lafayette, IN, USA) Wuppertal, Germany), Upper Extremity Functional Index, QuickDASH, Functional Assessment of Chronic Illness Therapy (FACIT) —Breast (FACT-B + 4)	At 12 months, patients treated with education alone presented more complex morbidity than the group treated with target physiotherapy. Target physiotherapy is therefore suitable for women with mammary carcinoma due to the early identification of upper limb morbidity
U. Olsson Möller I. Beck et al.	2019	Systematic review of systematic reviews to assess current evidence on rehabilitation in female patients following treatments for mammary carcinoma	37 systematic reviews of 1269 studies		There are strong positive effects in therapeutic exercise and in yoga for women treated for mammary carcinoma. Interventions with physical exercise improve shoulder joint movement, pain, lymphedema, fatigue and quality of life. Yoga instead influences the state of anxiety, depression, sleep disorders, gastrointestinal symptoms and quality of life.
Eduardo Castro-Martin, Lucia Ortiz- Comino et al.	2016	Investigate the immediate effect of myofascial induction related to electrotherapy as a control treatment on perceived pain, cervical-shoulder ROM and mood of patients who survived mammary carcinoma. It also examines the relationship between pain changes and the cervical and ipsilateral shoulder ROM to the mammary carcinoma.	Randomized single-blind study and placebo control. 21 cases diagnosed with mammary carcinoma stage I-IIIA and have completed adjuvant therapy (excluding hormone therapy)	VAS, goniometer, the Profile of Mood States and the Attitudes Towards Massage Scale.	A single session of myofascial induction reduces the intensity of pain and improves cervical-shoulder ROM significantly compared to the placebo group.
Hwang JH, Chang HJ, Shim YH, et al.	2008	Examine whether moderate-intensity supervised exercise can mitigate radiotherapy related complications	RCTs on 37 women treated with radiotherapy, of which 17 were in the supervised exercise group and 20 in the control group with exercises to perform at home	World Health Organization Quality of Life-BREF (WHOQOL-BREF), brief fatigue inventory (BFI), shoulder ROM and VAS	Statistically significant differences were found in changes in the WHOQOL, BFI, shoulder ROM, and pain scales

Discussion

Summary of Evidence

The present article subdivides shoulder pain in breast cancer survivors into three categories: musculoskeletal pain, neuropathic pain, of which the topic of drug-induced and radio-induced pain was examined, and finally chronic and central sensitization pain.

Nociceptive musculoskeletal pain, defined as a physiological response to an algogenic stimulus, is due to peripheral neuronal activity resulting from potentially harmful tissue stimuli [34]. Studies suggest treating it through joint and tissue mobilization techniques and manual therapy techniques with accessory joint mobilization and neurodynamics [35-36].

Neuropathic pain results from damage to peripheral or central nervous tissue, which causes chronic and self-sustained nerve stimulation, which results in altered responses of the somatosensory neurons. It is a burning, paroxysmal, electric shock pain and can be associated with paraesthesia, allodynia, or hyperalgesia [37]. Neuropathic pain may appear following treatment with radiotherapy: asthenia, skin reactions, and pain are, in fact, the most frequently reported side effects from radiotherapy [38]. Irradiation of the muscles and connective tissues within the shoulder joint can also lead to the formation of fibrosis and atrophy. Neuropathic pain can also occur following the use of hormonal therapy with aromatase inhibitors. This pain occurs in about 50% of women who survived breast cancer [38] and is described as widespread musculoskeletal pain, also called "aromatase inhibitor musculoskeletal syndrome or AIMSS." This pain persists even after stopping the drug. The side effects of aromatase inhibitors are, in fact, mainly at the expense of the osteoskeletal system with arthromial pain, osteoporosis, and risk of fractures. To address and reduce radio-induced and drug-induced neuropathic pain, both aerobic and reinforcement exercises have been proposed with the supervision of a physiotherapist to be performed twice weekly for a total of 150 minutes of exercise [22].

Chronic pain is defined as a pain present for at least six months and associated with profound changes in the personality and lifestyle of the patient that constitute maintenance factors independent of the action of nociceptors [39]. An inadequately managed pain in the postoperative phase is a significant risk factor in chronic pain syndrome, which occurs at a percentage that reaches 50% of patients at six months after surgery, as opposed to 12.7 % of women who have no history of breast cancer [40]. The treatment of chronic pain occurs through multidisciplinary management, including pain education interventions, pharmacological interventions, psychological interventions, and physiotherapy interventions [41].

Limitations

The present study contains several limitations. Among them, we include the scarce bibliography concerning the specific topic of shoulder pain following treatment for mammary carcinoma, although it is a very present theme on an epidemiological level. Further, only a few studies deal in depth with the methods of physiotherapy intervention carried out. Finally, the final selection of the articles chosen based on thematic correlation criterion was carried out by the same author, which compromises the objectivity of the study.

## Conclusions

Following surgery for breast cancer, multifactor physiotherapy was effective in the treatment of nociceptive musculoskeletal pain. It provides for tissue, accessory and neurodynamic joint mobilization plus active exercises with increasing intensity. After the sub-acute period, selective reinforcement exercises and neuromuscular proprioceptive facilitation are recommended for the restoration of the correct scapulohumeral rhythm. For drug-induced neuropathic pain, active exercise maintained for 12 months led to an improvement in arthralgia in previously inactive patients. The efficacy of the neurotaping used for five weeks was also observed, obtaining the subjective reduction of pain given by arthralgia associated with the use of anti-aromatase drugs. The most effective treatment for radio-induced neuropathic pain is a supervised and moderate-intensity therapeutic exercise. In addition to improving the pain sensation, statistically significant improvements in the WHOQOL, BFI, and shoulder ROM scales were calculated. Finally, in patients with chronic pain, multidisciplinary rehabilitation is useful to reduce disability and improve quality of life. This intervention is structured by combining psychological support for pain management and education, techniques for improving sleep problems, and active therapeutic exercise.
